# Distress and post-traumatic stress in parents of patients with congenital gastrointestinal malformations: a cross-sectional cohort study

**DOI:** 10.1186/s13023-022-02502-7

**Published:** 2022-09-11

**Authors:** D. Roorda, A. F. W. van der Steeg, M. van Dijk, J. P. M. Derikx, R. R. Gorter, J. Rotteveel, J. B. van Goudoever, L. W. E. van Heurn, J. Oosterlaan, L. Haverman, Emma Children’s Hospital Amsterdam UMC Follow-Me Program Consortium

**Affiliations:** 1 Amsterdam UMC, Emma Children’s Hospital, Department of Pediatric Surgery, Amsterdam Gastroenterlogy and Metabolism and Amsterdam Reproduction and Development research institutes, University of Amsterdam, Meibergdreef 9, 1105 AZ Amsterdam, The Netherlands; 2 Amsterdam UMC, Emma Children’s Hospital, Department of Pediatrics, Follow Me program and Emma Neuroscience Group, Amsterdam Reproduction and Development research institutes, University of Amsterdam, Amsterdam, The Netherlands; 3 Princess Maxima Center for Pediatric Oncology, Utrecht, The Netherlands; 4 Amsterdam UMC, Emma Children’s Hospital, Child and Adolescent Psychiatry and Psychosocial Care, Amsterdam Reproduction and Amsterdam Public Health research institutes, University of Amsterdam, Amsterdam, The Netherlands; 5 Amsterdam UMC, Emma Children’s Hospital, University of Amsterdam, Amsterdam, The Netherlands

**Keywords:** Distress, Post-traumatic stress disorder, Parental outcome, Surgery, Congenital malformations

## Abstract

**Background:**

Congenital gastrointestinal malformation (CGIM) require neonatal surgical treatment and may lead to disease-specific sequelae, which have a potential psychological impact on parents. The aim of this study is to assess distress and symptoms of post-traumatic stress disorder (PTSD) in parents of patients with CGIM. In this cross-sectional study, seventy-nine parents (47 mothers and 32 fathers) of 53 patients with CGIM completed the Distress Thermometer for Parents (DT-P) and the Self Rating Scale for Posttraumatic Stress Disorders (SRS-PTSD) as part of the multidisciplinary follow-up of their children (aged 5–35 months). Group differences were tested between parents and representative Dutch reference groups with regard to rates of (clinical) distress and PTSD, and severity of overall distress and PTSD, for mothers and fathers separately. Mixed model regression models were used to study factors associated with the risk of (clinical) distress, PTSD and with severity of symptoms of PTSD (intrusion, avoidance and hyperarousal).

**Results:**

Prevalence of clinical distress was comparable to reference groups for mothers (46%) and fathers (34%). There was no difference in severity of overall distress between both mothers as well as fathers and reference groups. Prevalence of PTSD was significantly higher in mothers (23%) compared to the reference group (5.3%) (OR = 5.51, *p* < 0.001), not in fathers (6.3% vs 2.2.%). Symptoms of intrusion were commonly reported by all the parents (75%). Longer total length of child’s hospital stay was associated with more severe symptoms of intrusion, avoidance and hyperarousal. Child’s length of follow-up was negatively associated with severity of intrusion.

**Conclusions:**

Having a child with CGIM has a huge impact on parents, demonstrated by a higher prevalence of PTSD in mothers, but not fathers, compared to parents in the general population. Monitoring of symptoms of PTSD of parents in follow-up is necessary.

**Supplementary Information:**

The online version contains supplementary material available at 10.1186/s13023-022-02502-7.

## Background

Congenital gastrointestinal malformations (CGIM) are birth defects of the gastrointestinal tract or abdominal wall (e.g., esophageal atresia, congenital diaphragmatic hernia, gastroschisis, omphalocele, intestinal atresia, Hirschsprung disease and anorectal malformations). CGIM require neonatal surgical treatment and hospital admission early in life and are associated with disease-specific chronic sequelae that require medical treatment at home, which may include: gastro-esophageal reflux and dysphagia or feeding problems requiring parenteral nutrition or gastrostomy in patients with congenital diaphragmatic hernia and esophageal atresia [1, 2], constipation or urinary/fecal incontinence in patients with gastroschisis, Hirschsprung disease and anorectal malformations [3–6], or the need for dilatations, rectal irrigation, and sometimes catheterization or an enterostomy in patients with Hirschsprung disease and anorectal malformations [7, 8]. This may have a psychological impact on their parents.

Parents of patients with CGIM may be vulnerable to psychological distress, because of the potentially traumatic effect of giving birth in general, but on top of that the unexpected and uncertain diagnosis, neonatal surgical treatment and disease-specific chronic sequelae their children may experience. Parents subsequently have complex responsibilities in taking care of the medical care of the child with CGIM, possibly in combination with the care for other siblings. Parents may thus frequently have experiences of loss of control, uncertainty and worries about admissions, treatment and prognosis of their children. All these experiences can be considered as potentially traumatic events, followed by short- or long-term stress responses. [9–19]. These repetitive stress responses can result in post-traumatic stress disorder (PTSD). [9].

Previous studies in parents of patients with CGIM have suggested that they are at risk of anxiety, depression, PTSD, impaired self-efficacy in home care [17, 20–22] and financial, practical, social problems [23–27]. However, previous studies often used small sample sizes and did not compare data on distress and PTSD with data from parents of healthy children. In addition, previous studies made no distinction between mothers and fathers, which may be of interest as studies focusing on other disorders identified significant sex differences in psychosocial experiences of parents [26, 28, 29]. As psychological distress in parents may influence parenteral quality of life and the parent–child interaction, it may subsequently also impact the wellbeing and functioning of the child [30]. We therefore integrated an online screening of parental psychological wellbeing in the protocols for multidisciplinary follow-up of patients with CGIM in our center.

In the current study, we aimed to assess parental (clinical) distress and PTSD symptoms in mothers and fathers of patients with CGIM in follow-up compared to data from mothers and fathers of Dutch reference populations using validated questionnaires. Parental distress in follow-up has not been studied before in parents of a pediatric surgical population. This is also the first study to assess parental distress and post-traumatic stress in fathers separately. Fathers are often underrepresented in studies that assess parental outcome. In addition, we aimed to study factors associated with (clinical) distress, PTSD and symptoms of PTSD.

## Methods

### Participants

In October 2017 a structured multidisciplinary follow-up program was implemented for patients with CGIM. In this program, follow-up visits are scheduled at the ages of 6 months, 12 months, 2, 6, 12, and 16 years of age. Before each follow-up visit, parents complete online questionnaires via the web-based Patient Reported Outcome Measures (PROM) portal KLIK (www.hetklikt.nu). Eligible for this study were parents of patients with esophageal atresia, gastroschisis, omphalocele, Hirschsprung disease, or anorectal malformations. Each parent with a child under the age of 36 months and visiting the follow-up program between implementation and March 2020, was included in this study. Parents were required to have sufficient skills in the Dutch language, and had to provide informed consent. In case parents visited the follow-up program multiple times, only the questionnaires completed at the first follow-up visit were used.

### Outcomes and measurements

#### Socio-demographic background of parents

Parental sex, age, educational level, employment status and parental country of birth were assessed via an online questionnaire administered via the patient portal of our electronic medical record system (EPIC MyChart). Parental educational level was scored on a 1–7 scale, and subdivided in low (0–3.5), middle (4 –5.5) and high (6–7) according to the Gold Standard 2017 (Statistics Netherlands, www.cbs.nl/en-gb). Employment status was defined as the number of parents of a couple that worked full-time (more than 36 h a week). Ethnic cultural background was expressed as the number of parents of a couple who were not born in the Netherlands.

#### Clinical characteristics of the patients

Type of malformation, length of postoperative hospital stay, and the child’s age at follow-up were extracted from the electronic medical record.

#### Parental distress

Parental distress was measured using the ‘Distress Thermometer for Parents’ (DT-P) [31]. The DT-P has been validated and showed acceptable internal consistency, and it’s diagnostic utility is examined in a large group of parents [31]. This scale contains 34 or 36 items, depending on the child’s age. It yields the following scores: an overall distress score (0 = no distress; 10 = extreme distress), for which a score of ≥ 4 is considered clinical relevant distress [32]. It further yields six problem domain scores (i.e., practical, social, emotional, physical, cognitive and parenting (an infant or a toddler)), as well as two total problem scores (with and without parenting problem domain score). The parenting domain has two versions (child < 2 and child > 2 years). The DT-P consist of three additional questions on the prevalence of chronic disease in parents, the experienced support from social surroundings and the need for psychosocial support. Data of a Dutch reference group of mothers and fathers is available (children aged < 36 months; 188 mothers, 141 fathers) [32].

#### Posttraumatic stress

PTSD was measured with the Self Rating Scale for Posttraumatic Stress Disorders (SRS-PTSD) questionnaire [33]. The SRS-PTSD has been validated and has shown good reliability, and high sensitivity (86%) and specificity (80%) compared to a structured clinical interview assessing PTSD [34]. The SRS-PTSD is a self-report questionnaire for adults and contains 22 items that correspond to the 17 symptoms of PTSD as described in the Diagnostic Statistical Manual of Mental Disorders (DSM), the fourth version. The SRS-PTSD consists of the three PTSD symptom domains: intrusion, hyperarousal and avoidance. All symptoms were scored on a four-point Likert scale (0–3), with scores of 2 or 3 reflecting the occurrence of a symptom and higher scores reflecting greater severity of symptoms. Intrusion was considered prevalent in case of at least one occurring intrusion symptom, avoidance was considered prevalent in case of at least three occurring avoidance symptoms and hyperarousal was considered prevalent in case of at least two occurring symptoms [33]. PTSD was considered prevalent when all three symptoms were prevalent. When filling out the SRS-PTSD, parents are asked to keep an event in mind that has had the most impact on them, and report on symptoms that occurred in the past four weeks. The sum of scores on all items was taken as a measure of severity of PTSD, and the sum of scores per symptom domain was taken as measure of symptom severity of intrusion, avoidance or hyperarousal. The rate of PTSD was compared to representative Dutch reference group (1141 females, 1097 males). [35]

### Statistical analysis

Statistical analyses were conducted using SPSS (version 25.0, SPSS Inc, Chicago, IL, USA) and R Studio (version 3.6.1, R Studio Team, PBC, Boston, MA, USA). Descriptive statistics were used to describe the characteristics of the included parents.

First, exploratory group comparisons were done between mothers and fathers of patients with CGIM and sex-matched parents of a Dutch reference group on the following outcome data: rate of clinically relevant distress, overall distress severity score, problem domain scores, total problem scores, answers to the additional questions on the DT-P, and rate of PTSD. In these group comparisons, independent *t*-tests (*t*) were used for normally distributed continuous data. For data that were not normally distributed, non-parametric testing was done with the Mann–Whitney U (*U*) test for the comparison of two groups and Kruskal Wallis test (*H*) for the comparison of multiple groups. For dichotomous data, *X2* test or Fisher’s Exact test were used based on the number of patients in each group. Standardized effect sizes were calculated to indicate the magnitude of the observed group differences (Cohen’s *d* for normally distributed continuous data, Rosenthal’s *r* for non-parametric continuous data and Odds Ratio (*OR*) for dichotomous data). Further exploratory analyses were done to assess on which specific items on the DT-P parents of patients with CGIM more often reported problems compared to a Dutch reference group.

Second, rate and severity of PTSD and PTSD symptoms were described separately for mothers and fathers within our sample. Furthermore, to assess covariance between answers of fathers and mothers of the same couple, correlations between the overall distress score and severity of PTSD severity score of fathers and mothers from the same couple within our sample were calculated.

Third, univariate and multivariate mixed model logistic and linear regression analyses were used to study risk factors for (clinical) distress, PTSD and symptom severity of distress, intrusion, and hyperarousal. Mixed model regression analyses were used to account for stronger associations between scores of parents of the same child. Only factors that were significantly associated with outcomes in univariate analysis were entered into multivariate analysis. The following possible risk factors were tested; sex of parent, average educational level of a couple, employment status of a couple, ethnic cultural background of a couple, type of child’s malformation, child’s age at follow-up, and length of in-hospital stay after surgery. Risk factors were excluded from analysis in case data were missing in more than 30% of the observations (which applied to the average educational level, employment status and ethnic cultural background of parents). In all analyses, an alpha-level of 0.05 was considered statistically significant.

## Results

### Parent and patient characteristics

The parents of a total of 111 patients were eligible for this study. The parents of 53 of 111 patients (48% response rate) were included. Of the 53 included families, both of the parents were included for 26 families and one of the parents was included for the remaining 27 families. This resulted in the inclusion of 79 parents (47 mothers and 32 fathers), of whom 78 parents responded to both questionnaires, and one parent only to the SRS-PTSD. Of the 79 parents, 64 parents were parenting an infant (< 2 year) and 15 were parenting a toddler (2–3 years). Sample characteristics of the mothers and fathers of patients with CGIM included in this study are listed in Table 1.

**Table 1:** Sample characteristics

	Parents of children with CGIM (*n* = 79)		
	All parents (*n* = 79)	Mothers (*n* = 47)	Fathers (*n* = 32)
Parental age in years, mean (SD) *	35.2 (5.7)	33.4 (4.6)	37.9 (6.2)
Child’s age at follow-up in months, median (range) *	8 (5–35)	8 (5–35)	9.5 (5–26)
Average educational level of parents per couple, median (range) * Low (1–3.5), *n* (%) Intermediate (4–5.5), *n* (%) High (6–7), *n* (%) *Missing, n (%)*	6 (4.5–7) 0 (0) 15 (19.0) 19 (24.1) 45 (57.0)	6 (4.5 – 7) 0 (0) 10 (21.3) 11 (23.4) 26 (55.3)	6 (5–7) 0 (0) 5 (15.6) 8 (25.0) 19 (59.4)
Number of parents with a country of birth other than the Netherlands per couple, *n* (%) 0 1 *Missing*	25 (31.6) 9 (11.4) 45 (57.0)	16 (34.1) 5 (10.6) *26 (55.3)*	9 (28.1) 4 (12.5) 19 (59.4)
Employment status per couple, *n* (%) 0 1 2 *Missing*	9 (11.4) 17 (21.5) 6 (7.6) 47 (59.5)	5 (10.6) 12 (25.5) 3 (6.4) 27 (57.4)	4 (12.5) 5 (15.6) 3 (9.4) 20 (62.5)
Number of responses per follow-up moment, *n* (%) 0.5 year 1 year 2 years	40 (50.6) 22 (27.8) 17 (21.5)	24 (51.1) 12 (25.5) 11 (23.4)	16 (50.0) 10 (31.3) 6 (18.7)
Child’s type of malformations, *n* (%) Esophageal Atresia Abdominal wall defects Hirschsprung disease Anorectal Malformations	26 (32.9) 13 (16.5) 22 (27.8) 18 (22.8)	15 (31.9) 9 (19.1) 12 (25.5) 11 (23.4)	11 (34.3) 4 (12.5) 10 (31.3) 7 (21.9)

^*^Normally distributed data is reported as mean (SD), and not normally distributed as median (range)

### Parental distress

In our sample, Cronbach’s alphas for the DT-P ranged from 0.70 to 0.87 for domain scores with the exception of the parenting > 2 years domain score (alpha = 0.31), and from 0.86 to 0.93 for total scores. The prevalence of clinically relevant distress in mothers (45.6%) and fathers (34.4%) of patients with CGIM was not significantly different compared to the sex-matched reference groups (47.9% and 34.8%, respectively). (Table 2). Similarly, overall distress severity scores, problem domain scores and total problem scores of both fathers and mothers on the DT-P did not differ from the sex-matched reference groups (Table 2 and Additional file 1: Table S1).

**Table 2:** Parental distress and post-traumatic stress in parents of patients with congenital gastrointestinal malformations (CGIM)

	Mothers CGIM *N* = 46	Normative mothers *N* = 188	Group difference, *p*-value	Effect size [95%CI]	Fathers CGIM *N* = 32	Normative fathers *N* = 141	Group difference, *p*-value	Effect size [95%CI]
Clinically relevant distress, *n* (%)	21 (45.6)	90 (47.9)	X^2^ = 0.07, *p* = .787	OR = 0,92 [0.84–1.75]	11 (34.4)	49 (34.8)	X^2^ = 0.002, *p* = .968	OR = 0.98 [0.44–2.21]
Overall distress scores, median (range)	3 (0–10)	3 (0–9)	U = 3980.5, *p* = .401	*r* = 0.05	2 (0–9)	2 (0–9)	U = 2119.0, *p* = .588	r = −0.04
*Problem domain scores with significant differences between parents of patients with congenital gastrointestinal malformations and normative data*								
Parenting < 2 years of age, median (range)	1 (0–7)	0 (0–6)	*U* = 2914.5, *p* = .006 *	*r* = 0.22	1 (0–7)	0 (0–5)	*U* = 1429.0, *p* = .072	*r* = 0.16
*Additional questions with significant differences between parents of patients with congenital gastrointestinal malformations and normative data*								
Wish to talk to professional, *n* (%)	20 (43.5)	32 (17.0)	X^2^ = 14.97, *p* < 0.001 *	OR = 3.75 [1.87–7.52]	6 (18.8)	20 (14.2)	X^2^ = 0.43, *p* = .584	OR = 1.40 [0.51–3.82]
*PTSD*								
PTSD,* n* (%)	11 (23.4)	60 (5.3)	X2 = 26.45, *p* < .001*	OR = 5.51 [2.67 – 11.35], *p* < .001 *	2 (6.3)	24 (2.2)	X2 = 2.00, *p* = .157	OR = 2.98 [0.67 – 13.19], *p* = .150

^*^Statistically significant, *p* < 0.05

On an item level, the following issues were more often reported by mothers compared to the reference group: problems in social interactions with friends, recurring thoughts about the health issues of their child, feelings of anxiety, problems with sexuality. And on the < 2 years parenting domain; problems with taking physical care of the children, problems with following up medical advice, and worries about the development of the children. Fathers of patients with CGIM more often reported issues with mood and (> 2 year) worries about the development of the children compared to the reference group. No differences were found on the additional questions, except for mothers reporting more often the wish to talk to a professional compared to the reference group. (Table 2). Overall distress scores in fathers and mothers of the same couple (26 couples) within our sample were not significantly correlated (*R* = 0.05, *p* = 0.723).

Univariate mixed model logistic regression showed that none of the four tested factors were significantly associated with the presence of clinical distress. Univariate mixed model linear regression showed that type of malformation was significantly associated with the overall distress severity score, whereas sex of the parent, child’s age at follow-up and length of child’s hospital stay were not.

### Posttraumatic stress

In our sample, Cronbach’s alpha for the SRS-PTSD ranged for each domain from 0.74 to 0.81 and was 0.89 for all items together. The prevalence of PTSD in parents of patients with CGIM was 16.5%. Mothers of patients with CGIM had a higher prevalence of PTSD (23.4%) compared to the reference group (5.3%) (*X*^*2*^ = 26.45, *OR* = 5.51, *95% CI:* 2.67 – 11.35, *p* < 0.001), whereas the prevalence in fathers of patients with CGIM was not significantly different compared to the reference group (Table 2).

Within our sample, 61 of the 79 parents reported symptoms in at least one domain of PTSD (77.2%). Prevalence of intrusion was reported in the great majority of parents of patients with CGIM (74.7%); respectively in 83.0% of the mothers and 62.5% of the fathers. Prevalence of avoidance was reported in 21.5% of parents; respectively in 27.7% of the mothers and 12.5% of the fathers. Prevalence of hyperarousal was reported in 27.8% of the parents; respectively in 38.3% of mothers and 12.5% of the fathers. Mean severity score of PTSD was 24 (22–58); respectively 25 (22–58) in the mothers and 24 (22–39) in fathers. Severity of PTSD in fathers and mothers of the same couple (*n* = 26 couples) in our sample was not significantly correlated (*R* = − 0.29, *p* = 0.841).

Univariate mixed model logistic regression showed that only male sex of the parent was negatively associated with the presence of PTSD. Univariate mixed model linear regression showed that male sex of the parent, child’s age at follow-up and that child’s length of hospital stay were associated with one of the symptom domains and were included in the multivariate analysis. The results of the multivariate mixed model regression models are shown in Table 3, and show that male parental sex was negatively associated with severity of intrusion, avoidance and hyperarousal, that child’s length of hospital stay was associated with severity of intrusion, avoidance and hyperarousal and that child’s age at follow-up was negatively associated with severity of intrusion.

**Table 3:** Risk factors for symptom severity of post-traumatic stress disorder in multivariate mixed model linear regression

	Severity of intrusion	Severity of avoidance	Severity of hyperarousal
	Multivariate Fixed effects, *ß* (95%-CI), *p*-value	Multivariate Fixed effects, *ß* (95%-CI), *p*-value	Multivariate Fixed effects, *ß* (95%-CI), *p*-value
*Type of parent*			
Mother	0 [Reference]	0 [Reference]	0 [Reference]
Father	−1.42 (− 2.46– −0.39), *p* = 0.009 **	−1.12 (−1.80 – −0.43), *p* = 0.003 **	−1.52 (−2.65– −0.38), *p* = 0.012 *
*Child’s age at follow-up*	−0.07 (− 0.14 – −0.01), *p* = 0.037 *	NA	NA
*Child’s length of hospital stay*	0.03 (0.01 – 0.06), *p* = 0.009 **	0.03 (0.01 – 0.05), *p* = 0.021 *	0.03 (0.004 – 0.06), *p* = 0.032 *

^*^ *p* < 0.05, ** *p* < 0.01, *** *p* < 0.001

## Discussion

### Summary of findings

This study aimed to assess distress and PTSD in parents of patients with CGIM, using data from clinical screening in follow-up, whilst comparing mothers to mothers and fathers to fathers from reference groups and using validated questionnaires. This has not been described previously by other studies.

With regard to parental distress, our findings showed that parents of patients with CGIM experience equal amounts of parental distress compared to reference groups, which is in line with other studies assessing distress in parents of patients with esophageal atresia and anorectal malformations [10, 12]. However, some specific problems were reported more often by parents, including problems with parenting, concerns about the development of children, and in mothers emotional problems, problems with social interactions and recurring thoughts about the admission in mothers, in line with higher levels of PTSD in mothers. Mothers also more often expressed the wish to talk to a professional compared to the reference group.

With regard to parental post-traumatic stress, our findings showed that parents of patients with CGIM are at risk for developing PTSD symptoms and that almost a quarter of the mothers met the criteria for PTSD. Our findings further showed that from the three major PTSD symptoms (intrusion, avoidance and hyperarousal), intrusions were most commonly reported. Intrusions are sudden recalls or nightmares of a certain traumatic experience, that initiate an acute stress response [36]. Also in case no diagnosis of PTSD has been made and intrusions occur secluded from others symptoms of PTSD, intrusions can cause an acute stress response which in turn can contribute to chronically elevated stress levels [9]. Our findings showed that the prevalence and risk of PTSD was higher in mothers with CGIM but not in fathers, compared to Dutch reference groups. This sex difference has been reported more often in previous studies and may be related to stronger perceptions of threat and loss of control in women [28, 37, 38]. Our findings that mothers of patients with CGIM are at risk for PTSD are in line with a previous study of Le Gouez et al. in parents of patients with esophageal atresia. In that study even higher rates of PTSD were found, and PTSD was found both in fathers and mothers [11]. Le Gouez et al. had included younger patients and only patients with esophageal atresia, which may explain differences. Although we expected that differences in child’s type of malformation would explain differences in severity of distress and PTSD symptoms, because of differences in the treatment and disease-specific sequelae between different types of malformations, our findings did not suggest this [12, 24]. The factors that induce a posttraumatic stress response may also be related to premorbid psychological wellbeing of parents, including previous episodes of PTSD, pre-existent personality disorder, depression or anxiety disorder [36]. Other inducing factors may be related to receiving an unexpected diagnosis, general aspects of medical treatment and hospital admission, including communication by health care professionals, that attribute to experiences of uncertainty or experiences of loss of control [15, 18, 39]. These factors may contribute to developing PTSD regardless of the child’s type of malformation, as this is highly dependent on the subjective interpretation of parents rather than the objective information provided. Our findings did suggest that length of child’s hospital stay was associated with increased severity of PTSD symptoms. This suggests that being in an environment with stress-inducing stimuli may play a role and create recurrent and stacked stress responses, thus adding to more severe symptoms of intrusions, avoidance and hyperarousal. Previous evidence also shows that giving birth may be a traumatic experience in itself [40–42]. Mothers of patients with a birth defect may have an increased risk, as among the risk factors for traumatic childbirth are psychological difficulties during pregnancy, obstetric or infant complications, and emergency caesarean section. CGIM may be associated with a prenatal diagnosis inducing psychological distress during pregnancy, delivery by caesarean section and infant complications after delivery. [43]

Our findings emphasize the need to pay attention to parents during follow-up of patients with CGIM. Although collecting Patient Reported Outcome Measure (PROM) data in clinical practice has it challenges, our study shows the relevance and usefulness screening of parents for issues in daily life that cause distress and symptoms of PTSD. Routine monitoring of PROM data can help to early recognize problems in parental psychological wellbeing. Early recognition of psychological distress and PTSD symptoms may lead to interventions. Evidence shows that Eye Movement Desensitization and Reprocessing therapy (EMDR) has good effects on PTSD symptoms, particularly intrusions [44]. All parents who experience intrusions, not just those with an official diagnoses of PTSD, may benefit from EMDR treatment. Other possible interventions that parents may benefit from are social work, parental support groups, or social media support groups [45–47]. Interventions may prevent further negative consequences of psychological distress and PTSD symptoms from occurring, including disrupted bonding between parents and child, difficulties in the relationship between parents and problems with parenting, which in turn may have negative impact on family functioning and child’s functioning [10, 48–50]. Moreover, PTSD is associated with higher risk of anxiety and depression disorder [41, 51], and may lead to increased absenteeism from work or social activities. [52]

### Limitations

Our findings need to be interpreted in the light of a few limitations that were part of this study. First, there was a risk of inclusions bias resulting in low response rates. We collected PROM data in a multidisciplinary program that was implemented during the study period. Response rates may have been negatively influenced by challenges in the implementation of the multidisciplinary follow-up, including difficulties of parents with the activation of their account on the online portal for questionnaires. Moreover, completing questionnaires can be time-consuming, thus decreasing the response rate. Because we used standardized questionnaires for our outcome measurement, there was no possibility to qualitatively assess aspects of distress and intrusions. Secondly, our sample size, the amount of missing data on socio-demographic characteristics, as well as the exclusion of parents with a limited ability of the Dutch language, limited the possibility to assess factors associated with the risk and symptom severity of parental distress and PTSD. Lastly, because of the number of comparisons done in this study, and the exploratory nature of some of the analyses, the risk of chance findings cannot be ruled out. In particular the findings from the comparison on an item-level of the DT-P between parents of patients with CGIM and references groups thus require caution and await further replication in larger samples.

### Future perspectives

We think future studies should focus on identification of factors that contribute to distress and PTSD symptoms in parents of patients with CGIM, including socio-demographic characteristics, premorbid psychopathology, previous traumatic experiences, and aspects of medical treatment or communication by health care professionals. Studies should also focus on how to empower factors that may protect parents of patients with CGIM from developing PTSD, including coping skills, psychoeducational support during hospital stay, and supportive communication by health care providers.

For future studies it would further be interesting to use a qualitative approach with interviews or focus groups to assess the nature of the intrusions parents experience and what triggers intrusions. This may provide more insight in the specific experiences that are traumatic to parents. Insight in factors that contribute to distress and PTSD may lead to interventions to prevent distress and PTSD.

Lastly, we recommend longitudinal monitoring of psychological wellbeing of parents in follow-up of patients with CGIM. This allows for the assessment of longitudinal trends in distress and PTSD symptoms and may provide more insight in the type of PTSD the parents may experience, triggers for intrusion and the effects of interventions, including EMDR, psychoeducational interventions, parent-support groups and online interventions. [44–47, 53].

Attending pediatric surgeons should be aware of the stressful and potentially traumatic effects of the treatments they provide on the parents of their patients, strive for open and frequent communication in order to lower stress levels in parents. Routine monitoring of PROM data can help to early recognize parents that may benefit from interventions that help them process their experience. This shows why providing standardized follow-up by a multidisciplinary team including a psychologist, should be recommended for all expertise centers treating surgical congenital malformations.

## Conclusions

In this study we demonstrated a higher prevalence of PTSD in mothers of patients with CGIM compared to a reference group and symptoms of intrusion in a majority of parents. These findings emphasize the need for monitoring of psychological wellbeing of parents of patients with CGIM in follow-up and that screening on PTSD symptoms may contribute to integrative, family-centered care. Early recognition of PTSD or distress symptoms and early interventions may improve psychological wellbeing of the parents.

## Supplementary Information

**Additional file 1: Table S1** Non-significant differences in parental distress scores in parents of patients with congenital gastrointestinal malformations (CGIM) compared with normative data.

## Data Availability

The datasets used and/or analysed during the current study are available from the corresponding author on reasonable request.
