# Supplementary material for: Distress and post-traumatic stress in parents of patients with congenital gastrointestinal malformations: a cross-sectional cohort study
Source: Orphanet J Rare Dis. 2022 Sep 11;17:353. doi: 10.1186/s13023-022-02502-7 (PMC9465926; doi:10.1186/s13023-022-02502-7)
Supplement: Supplementary file 1 — Additional file 1: Table S1 Non-significant differences in parental distress scores in parents of patients with congenital gastrointestinal malformations (CGIM) compared with normative data. [file 13023_2022_2502_MOESM1_ESM.docx]

**sTable 1** Non-significant differences in parental distress scores in parents of patients with congenital gastrointestinal malformations compared with normative data

| **Problem domain scores that were not significantly different between parents of patients with congenital gastrointestinal malformations and normative data** | | | | | | | | |
| --- | --- | --- | --- | --- | --- | --- | --- | --- |
|  | Mothers CGIM  N=46 | Normative mothers N=188 | *Group difference, p-value* | *Effect size [95%CI]* | Fathers CGIM  N=32 | Normative fathers  N=141 | *Group difference, p-value* | *Effect size [95%CI]* |
| Practical problem score *median (range)* | 1 (0-7) | 1 (0-7) | U=4108.0, p=.587 | r=-0.04 | 0 (0-4) | 1 (0-7) | U=1973.0, p=.236 | r=-0.09 |
| Social problem score, *median (range)* | 0 (0-4) | 0 (0-3) | U=4580.0, p=.421 | r=0.05 | 0 (0-3) | 0 (0-2) | U=1897.5, p=.057 | r=-0.14 |
| Emotional problem score, *median (range)* | 1 (0-9) | 1 (0-9) | U=4686.5, p=.363 | r=0.06 | 0 (0-4) | 0 (0-9) | U=1829.5, p=.065 | r=-0.14 |
| Physical problem score, *median (range)* | 2 (0-7) | 2 (0-7) | U=4485.5, p=.690 | r=0.03 | 1 (0-5) | 1 (0-6) | U=2121.5, p=.587 | r=-0.04 |
| Cognitive problem score, *median (range)* | 0 (0-2) | 0 (0-2) | U=4731.0, p=.262 | r=0.07 | 0 (0-2) | 0 (0-2) | U=2364.0, p=.559 | r=0.04 |
| Parenting ≥ 2 years of age, *median (range)* | 0 (0-2) | 0 (0-3) | U=178,5, p=.065 | r=-0.03 | 0 (0-1) | 0 (0-3) | U=157.5, p=.928 | r=-0.02 |
| **Total problem scores that were not significantly different between parents of patients with congenital gastrointestinal malformations and normative data** | | | | | | | | |
| Total problem score, *median (range)* | 6 (0-24) | 6 (0-24) | U=4517.5, p=.637 | r=0.03 | 2 (0-17) | 3 (0-22) | U=1836.0, p=.098 | r=-0.13 |
| Total with <2 years parenting, *median (range)* | 9 (0-25) | 7 (0-27) | U=2699.5, p=.102 | r=0.13 | 3 (0-24) | 3 (0-23) | U=1045.5 p=.356 | r=-0.08 |
| Total with ≥2 years parenting, *median (range)* | 2 (0-16) | 6 (0-24) | U=178,5, p=.065 | r=-0.22 | 3 (0-10) | 3 (0-19) | U=138.5, p=.562 | r=-0.08 |
| **Additional questions that were not significantly different between parents of patients with congenital gastrointestinal malformations and normative data** | | | | | | | | |
| Support from surroundings, *n (%)* | 42 (91.3) | 171 (91.0) | X^2^=0.01, p=.941 | OR=1.04 [0.33-3.27] | 29 (90.6) | 129 (91.5) | X^2^=0.03, p=.875 | OR=0.89 [0.24-3.39] |
| People show lack of understanding, *n (%)* | 6 (13.0) | 30 (16.0) | X^2^=0.41, p=.623 | OR=0.79 [0.31-2.03] | 2 (6.3) | 15 (10.6) | X^2^=0.57, p=.742 | OR=0.56 [0.12-2.58] |
| Parent has chronic illness, *n (%)* | 7 (15.2) | 44 (23.4) | X^2^=1.45, p=.228 | OR=0.59 [0.25-1.41] | 4 (12.5) | 16 (11.3) | X^2^=0.03, p=0.768 | OR=1.12 [0.35-3.60] |
